# Robust pathway sampling in phenotype prediction. Application to triple negative breast cancer

**DOI:** 10.1186/s12859-020-3356-6

**Published:** 2020-03-11

**Authors:** Ana Cernea, Juan Luis Fernández-Martínez, Enrique J. deAndrés-Galiana, Francisco Javier Fernández-Ovies, Oscar Alvarez-Machancoses, Zulima Fernández-Muñiz, Leorey N. Saligan, Stephen T. Sonis

**Affiliations:** 1 Group of Inverse Problems, Optimization and Machine Learning, Department of Mathematics, University of Oviedo, C/ Federico García-Lorca, 18, 33007 Oviedo, Spain; 2 Department of Informatics and Computer Science, University of Oviedo, C/ Federico García-Lorca, 18, 33007 Oviedo, Spain; 3 National Institutes of Health, National Institute of Nursing Research, Bethesda, MD USA; 4 Primary Endpoint Solutions, Watertown, MA USA; 5 Brigham and Women’s Hospital and the Dana-Farber Cancer Institute, Boston, MA USA

## Abstract

**Background:**

Phenotype prediction problems are usually considered ill-posed, as the amount of samples is very limited with respect to the scrutinized genetic probes. This fact complicates the sampling of the defective genetic pathways due to the high number of possible discriminatory genetic networks involved. In this research, we outline three novel sampling algorithms utilized to identify, classify and characterize the defective pathways in phenotype prediction problems, such as the Fisher’s ratio sampler, the Holdout sampler and the Random sampler, and apply each one to the analysis of genetic pathways involved in tumor behavior and outcomes of triple negative breast cancers (TNBC). Altered biological pathways are identified using the most frequently sampled genes and are compared to those obtained via Bayesian Networks (BNs).

**Results:**

Random, Fisher’s ratio and Holdout samplers were more accurate and robust than BNs, while providing comparable insights about disease genomics.

**Conclusions:**

The three samplers tested are good alternatives to Bayesian Networks since they are less computationally demanding algorithms. Importantly, this analysis confirms the concept of “biological invariance” since the altered pathways should be independent of the sampling methodology and the classifier used for their inference. Nevertheless, still some modifications are needed in the Bayesian networks to be able to sample correctly the uncertainty space in phenotype prediction problems, since the probabilistic parameterization of the uncertainty space is not unique and the use of the optimum network might falsify the pathways analysis.

## Background

Phenotype prediction is one of the forefront challenges in the drug design industry; a problem that consists of finding the set(s) of genes that affects pathogenesis. Computationally speaking, this type of prediction problem is ill-posed, since the number of supervised genetic probes always exceeds the number of samples. In this sense, a large and vast uncertainty space associated to this problem is found, thus; characterizing the involved biological pathways is an ambiguous task, mainly due to the existence of equivalent genetic networks that may lead to a phenotype prediction with similar accuracies [1, 2].

Moreover, one of the major difficulties in the study of genetic data is the lack of a theoretical model that associates different genes/probes to a class prediction. Mathematically speaking, this consists of an operator that given a set of genetic signatures **g** it is possible to predict a set of classes, C = {1, 2}, of the phenotype:

1 $$ {\mathbf{L}}^{\ast}\left(\mathbf{g}\right):\kern0.33em \mathbf{g}\kern0.33em \in \kern0.33em {\mathbb{R}}^{\mathrm{s}}\to \mathrm{C}\kern0.33em =\kern0.33em \left\{\mathbf{1},\kern0.33em \mathbf{2}\right\} $$

The simplest case is to divide the phenotype in healthy controls and disease samples, but others problems concerning drug optimization can be casted into this framework.

By optimizing the cost function, O(**g**) = ‖**L**^∗^(**g**) − **c**^obs^‖_1_, that measures the distance between the observed classes in the training dataset of data (**c**^obs^) and the associated set of predictions **L**^∗^(**g**), via the genetic signature **g** and the classifier **L**^∗^, it is possible to find the set of discriminatory genetic signatures. In this notation ‖**L**^∗^(**g**) − **c**^obs^‖_1_ represents the amount of uncorrected samples predicted by the classifier. Therefore, the accuracy of a genetic signature is: Acc(**g**) = 100 − O(**g**).

The uncertainty space relative to **L**^∗^, M_tol_ = {**g** : O(**g**) < tol}, is formed by the groups of high predictive networks that have a similar predictive accuracy, Acc(**g**). These networks are located in one or several flat curvilinear valleys of the cost function topography, O(**g**) [3, 4], concerning the classifier **L**^∗^(**g**).

This research is based on two main hypothesis: 1. the high discriminatory genetic networks located in M_tol_ serve to understand the reasons behind disease development in order to discover alternative therapeutic targets. 2. These biological pathways should be independent of the classifier and of the sampling method used to unravel them. This is named the hypothesis of biological invariance.

In this paper, we compare different sampling methods to establish a robust identification of the altered genetic pathways in a disease. The first method is the Fisher’s ratio sampler [5] that explores the defective pathways considering the discriminatory capacity of the differentially expressed genes according to their Fisher’s ratio that provides the “a priori” sampling distribution of the high-discriminatory networks. The second sampling algorithm, known as Holdout sampler, is inspired by the bootstrapping technique [6, 7]. This algorithm quantifies the likelihood of the high discriminatory genetic networks using k-NN classifier in a validation data set using the minimum-scale genetic signature found in the training set of each random holdout. In this case, the “a priori” probability distribution is established by the discriminatory capability of the different networks in the training dataset in each random hold out (minimum-scale genetic signature). Therefore, this algorithm is based on a complete different sampling paradigm than the Fisher’s ratio sampler. One of its main features is its fatness and robustness in assessing the uncertainty of the solution of inverse and regression problems [8, 9]. The third methodology consists of randomly sampling the set of differentially-expressed genes in the phenotype. This algorithm selects random-wise genes within this set with a prior uniform distribution, building genetic signatures of different lengths (number of genes) between some bounds that are related to the complexity of the phenotype prediction problem, that is, the minimum number of genes with the highest predictive accuracy (or minimum-scale signature). For these three samplers (Fisher’s, Holdout and Random), the signatures that have been sampled and better predicted the phenotype, are used in the posterior frequency analysis of the discriminatory genes, that serves to establish the ontological pathway analysis. Finally, the last procedure is based on Bayesian Networks (BN), a popular predictive modeling formalism in bioinformatics, with many applications in modern genomics [10–13].

## Materials

These sampling methodologies were applied to a microarray dataset obtained from Gene Expression Omnibus concerning the Triple Negative Breast Cancers (TNBC) phenotype to unravel the altered genetic pathways that control metastasis and survival in this type of aggressive cancers. This dataset was first analyzed by Jézéquel et al. [14], and can be accessed in the Gene Expression Omnibus (GEO) under the acronym GSE58812. This microarray comprises the gene expressions of 107 patients with TNBC and controlled for metastasis (44 relapsed and 63 were disease-free after a follow-up period of 7 years) and survival (78 survived and 29 were deceased during the control period). These patients were treated between 1998 and 2007 at the Institut de Cancérologie de lOuest – René Gauducheau and the Institut de Cancérologie de lOuest – Paul Papin. This data received the consent of patients as required by the French Committee for the Protection of Human Subjects (CCPPRB). The analysis of the gene expression was carried out in quality control RNA samples by Affymetrix® Human Genome U133 Plus 2.0 Arrays (Affymetrix®, Santa Clara, CA, USA), and the microarray analysis measured over 43,000 transcripts. This type of cancer was selected due to its high metastatic potential and very low prognosis rates (survival). However, this procedure can be applied to the study of other diseases using genetic data.

## Methods

### Feature selection

A previous gene filtering according to their discriminatory power was performed for all the sampling algorithms. Fold-change analysis served to detect those genes that were differentially expressed (over and under-expressed genes). Furthermore, the idea of performing fold-change analysis is to enhance the sampling of the header genes, which are those that outline the most important features of the phenotype prediction [15]. The rest are helper genes that explain high frequency details of the discrimination. This procedure is similar to the Fourier decomposition of a signal into its harmonics. It is of utmost importance to understand the ill-posed character of the phenotype prediction problem, due to the fact that the number of genetic probes that are monitored are much higher than the number of samples, making the uncertainty space very vast and difficult to sample. Furthermore, the irruption of next generation DNA sequencing (NGS) techniques makes this imbalance even greater. As it has been already outlined, the genetic data used in this paper comes from the gene expression of the transcriptome. Nevertheless, although the analysis of the transcriptome involve the acquisition of smaller amount of genetic information than NGS, the number of monitored genetic probes still exceeds more than 400 times the number of diagnosed samples. This fact clearly outlines the need of gene filtering techniques to reduce the dimension of the set of genes that might be related to the disease development.

### Bayesian approach of uncertainty

The Bayesian approach of uncertainty in phenotype prediction problems consists of sampling high discriminatory genetic networks (**g**) of the phenotype according to Bayes rule:

2 $$ \mathrm{P}\kern0.33em \left(\mathbf{g}/{\mathbf{c}}^{\mathrm{obs}}\right)=\mathrm{P}\kern0.33em \left(\mathbf{g}\right)\kern0.33em \mathrm{L}\left({\mathbf{c}}^{\mathrm{obs}}/\mathbf{g}\right)/\kern0.33em \mathrm{P}\left({\mathbf{c}}^{\mathrm{obs}}\right), $$

where P(**g**/**c**^obs^) is the posterior distribution of the genetic signature **g**, P(**g**) is its prior sampling distribution, L(**c**^obs^/**g**) is the likelihood, that depends on the predictive accuracy of the genetic signature, *Acc*(**g**) = 100 − O(**g**), and P(**c**^obs^) is called the evidence of the observed classes. A genetic signature has a bigger likelihood if the probability of observing the class array (**c**^obs^) is bigger, that is, the prediction error, O(**g**), smaller.

The analytical expression of P(**g**/**c**^obs^) is unknown. Therefore, different sampling algorithms are needed to infer diverse genetic networks from the high probability region of P(**g**/**c**^obs^) in order to understand the phenotype from the mechanistically point of view. The aim of this comparison is to show that the sampled networks using different algorithms are mechanistically similar. This fact would be a confirmation that we are achieving a correct sampling of the defective pathways and enforcing the hypothesis of biological invariance, which states that these pathways should be independent of the algorithm and the classifier used to perform their sampling.

### Sampling algorithms

#### Fisher’s ratio sampler (FRS)

FRS is a fast and robust sampling algorithm. The *FRS* weighs the discriminatory power of the expressed genes by quantifying its Fisher’s ratio in order to obtain an “a priori” sampling distribution of high discriminatory genetic network. The sampled networks are random-wise established using this pre-defined distribution, while its likelihood is determined via Leave-One-Out-Cross-Validation (LOOCV) using a nearest-neighbor classifier [15].

**Fig. 1:**
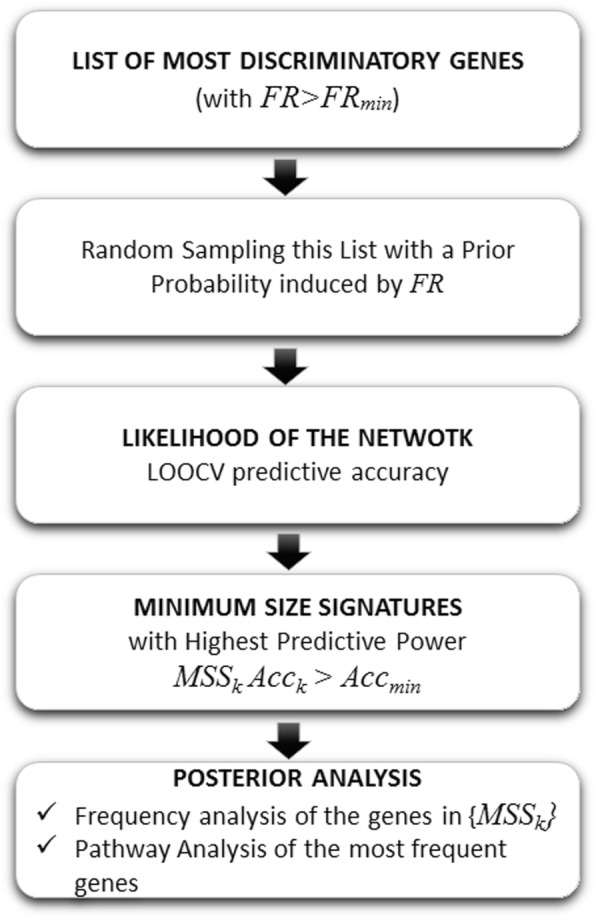
Fisher’s ratio sampler workflow

The algorithm workflow (Fig. 1) is as follows:

- The set of genes with the highest Fisher’s ratio is identified from the set of genes with the highest fold change. To this end, differentially expressed genes (over and under-expressed) were found and ranked according to their Fisher’s ratio in order to detect those genes that homogeneously separate within classes (low-intra class variance). In a binary classification problem the Fisher’s ratio of the gene *j* is:

3 $$ F{R}_j=\frac{{\left({\mu}_{j1}-{\mu}_{j2}\right)}^2}{\sigma_{j1}^2+{\sigma}_{j2}^2}, $$

where *μ*_*ji*_ is a measure of the center of mass of the probability distribution of the gene *j* in class *i*, and *σ*_*j*i_ is a measure of its dispersion within this class. Discriminatory genes have a high Fisher’s ratio since they have a low-intra class dispersion and high inter-class distance, which informs us about the separation between the centers of the corresponding prognostic genes distributions. In this paper, Discriminatory genes are defined as those that are differentially expressed with a Fisher’s ratio greater than *f*_*r*_ = 0.8, that is, the hubs of the distribution in both classes are separated: $\left|{\mu}_{j1}-{\mu}_{j2}\right|>0.89\sqrt{\sigma_{j1}^2+{\sigma}_{j2}^2}$. The Fisher’s ratio cutoff value could be further decreased to *f*_*r*_ = 0.5 if the number of discriminatory genes within this set is very low. Therefore, the Fisher’s ratio cut-off value is an important tuning parameter in this procedure.

Finding the minimum-scale genetic signature. Based on the quantified Fisher’s ratio, the discriminatory genes are ranked in a descendent order, then; the algorithm is capable of detecting the minimum-scale signature, which better separates the classes via recursive feature elimination. The predictive accuracy estimation is based on LOOCV, utilizing a nearest-neighbor classifier and recursive feature elimination [1, 15]. The minimum-scale signature serves to estimate the length (number of genes) of the high discriminatory networks to be sampled.

**Fig. 2:**
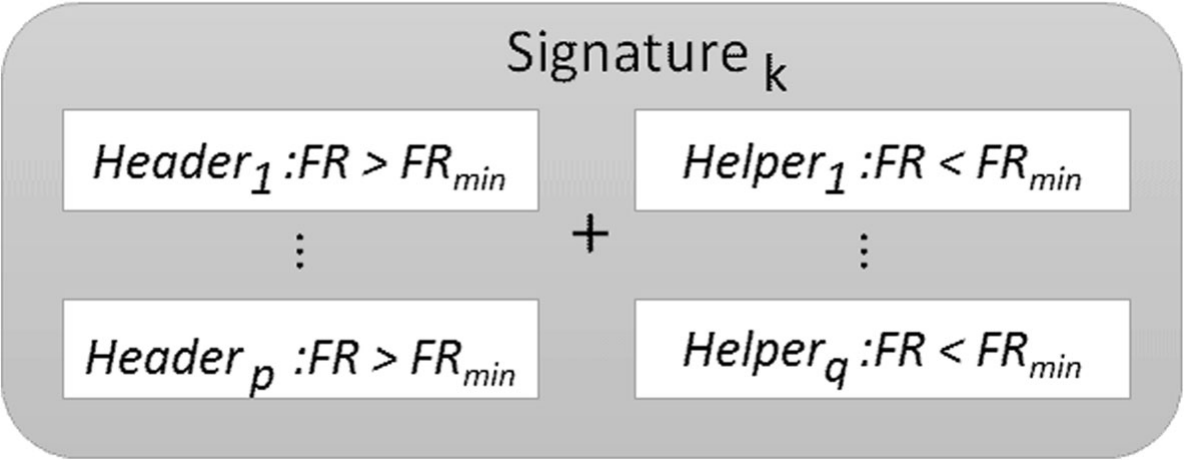
Genetic Network built by the FRS at step k

- Random sampling of high discriminatory equivalent networks. By randomly sampling, it is possible to find out other discriminatory networks using a prior sampling probability of any individual gene proportional to its Fisher’s ratio. Genes are rated as Headers (genes with *FR*_*j*_ > *FR*_*min*_) and Helpers (*FR*_*j*_ < *FR*_*min*_), as shown in Fig. 2, and the Fisher sampler constructs genes signatures at each step, by selecting some Headers and some Helpers that meet the following conditions:
  - *max(cdf (Headers) < rand(1))*
  - *max(cdf (Helpers) < rand(1));*
  where cdf is the empirical cumulative distribution function in the sets of genes Headers and Helpers sets respectively, and rand(1) is a random number between 0 and 1.

In this sampling algorithm, high discriminatory variables span the most important features of the classification while lower discriminatory variables account for discrimination details. This method minimizes the high-frequency details (helper genes) while optimally discriminating between classes and promoting the header genes, which are those that explain the phenotype in a robust manner. It is therefore expected that different associations of headers and helper genes form the high discriminatory genetic networks. It is important to remark that only with helper genes a high discrimination accuracy cannot be achieved. The LOOCV predictive accuracy is calculated after a genetic network has been randomly constructed based on the Fisher’s probability distribution.

FRS follows the Bayes rule (2) with a prior probability −P(**g**) − inferred from the Fisher’s ratio of the selected genes and a likelihood −L(**c**^obs^/**g**)− that depends on the LOOCV predictive accuracy of the genetic network **g** that has been sampled. However, it is out the scope of this paper to explore the posterior distribution factorization, but to determine the genes with the highest discriminatory power that are in relation with the uncertainty space of the problem [1, 3, 4] via **L**^∗^(**g**) in order to identify the altered genetic pathways.

Finally, the discriminatory networks with the highest posterior sampling frequency are those that best defined the TNBC phenotype. A frequency threshold is used to optimize the discriminatory genes used for the pathway analysis. Based on these networks the defective biological pathways are identified via Gene Analytics [16].

A simplified version of this algorithm has been previously used to assess the genomic risk of aromatase inhibitor-related arthralgia in patients with breast cancer using SNPs [17], to perform the integration of genomic data in CLL patients [18, 19], and to predict post-radiotherapy fatigue development in cancer patients [20].

#### Holdout sampler (HS)

The rationale of the HS algorithm is completely different from FRS; however, the purpose is the same: exploring the uncertainty space intrinsic to phenotype prediction problems. In this sense, the procedure consists of changing the evidence term of the observed classes - P(**c**^obs^) - in Bayes expression (2). The simplest way of doing that is performing random data bags with different datasets for training, followed by a blind validation. This is comparable to modifying the evidence of **c**^obs^ with respect to the classifier **L**^∗^, since part of the samples used for blind validation have not been used (observed in training). This method is grounded on the statistical technique of bootstrapping, or arbitrary sampling with replacement [7], which is used to build the confidence intervals in sample estimates and to estimate the sampling distribution of any statistic via a random sampler. In this case, this methodology was designed to explore the uncertainty space in phenotype prediction. This algorithm was used in other disciplines and fields of technology to optimally sample the model parameters posterior distribution via the least squares fitting of different data bags [6, 8, 9].

**Fig. 3:**
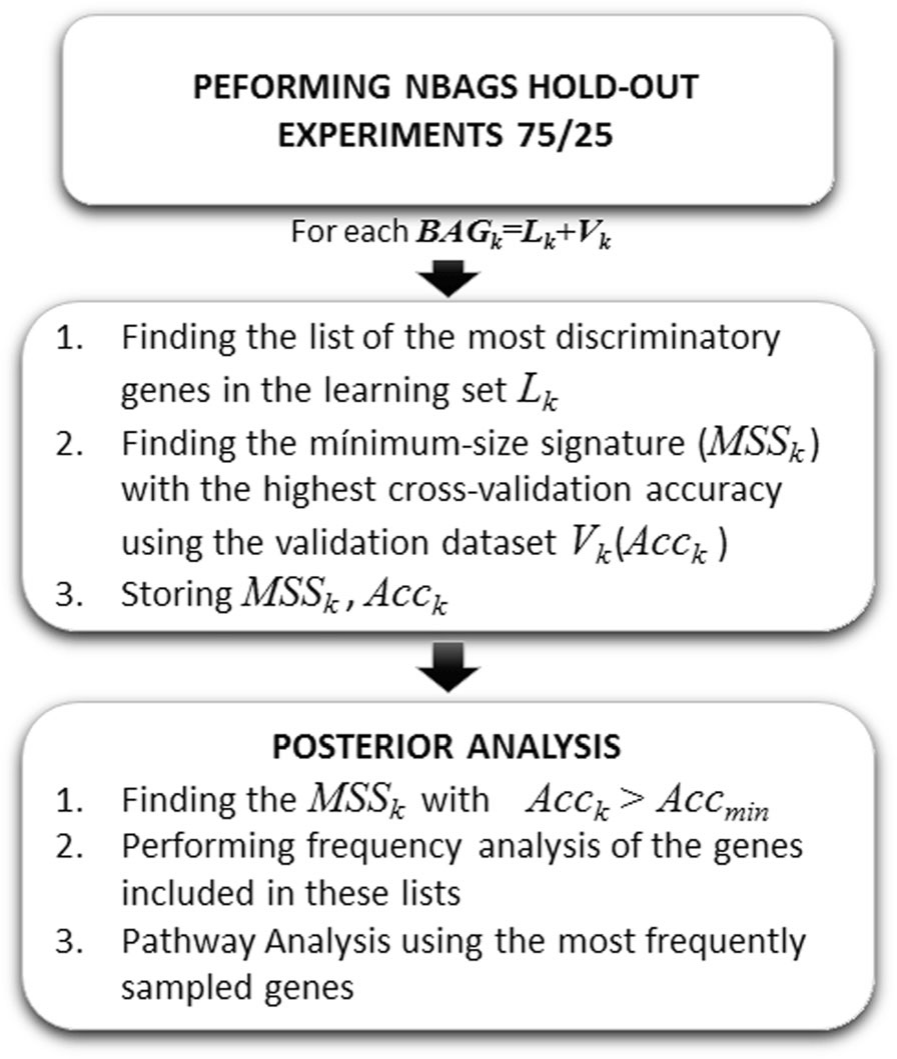
The Holdout Sampler workflow

Figure 3 shows the HS workflow. This algorithm samples the uncertainty space in two steps:

- Data *bagging:* Different arbitrary 75/25 data bag holdouts were different from the dataset, where 75% of the data is used for learning and 25% for validation. In the present case, 1000 different bags were generated. For each bag, the minimum-scale signature is established using the training dataset following the same procedure than for FRS, and the overall predictive accuracy estimation is established via LOOCV over all the samples of the validation dataset in each bag. Therefore, in the case of HS the sampling consists in finding the minimum scale signature using the training of the data bag and establishing its likelihood in the validation part via LOOCV. The holdout sampler involves a k-NN classifier in the reduced set of high discriminatory genes (minimum-scale signature) which has been successfully applied to the bioinformatics modeling of high dimensional Omics data [15, 18].
- Posterior analysis: after completing the bags simulation, the posterior analysis is carried using the minimum-scale signatures that have been sampled, having a LOOCV validation predictive accuracy above a given threshold. In this case an accuracy threshold of 85% was found to provide enough explicative genetic networks of the TNBC phenotype. The accuracy threshold is tuning parameter of the procedure that depends on the maximum predictive accuracy that can be achieved.

Finally, these lists follow a frequency analysis to find the most frequently sampled genes required to establish the defective genetic pathways via ontological platforms.

#### Random sampler (RS)

**Fig. 4:**
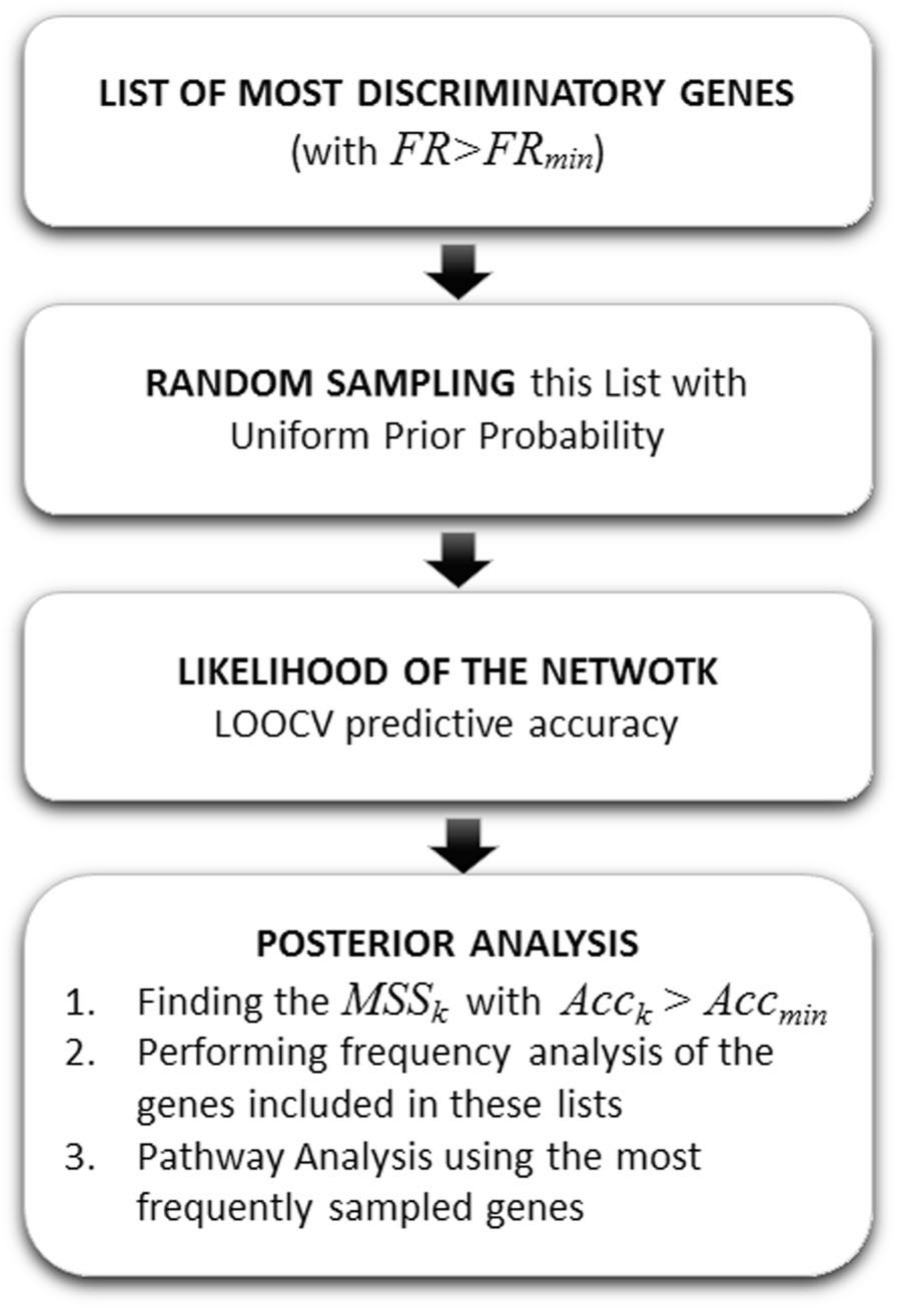
The Random Sampler workflow

This algorithm randomly selects genes and builds signatures of variable length [21]. The philosophy is close to the FRS, however, in this case the “a priori” sampling distribution is uniform instead of proportional to the Fisher’s ratio, As in the FRS algorithm, the predictive accuracy is established via LOOCV. The posterior frequency analysis and the ontological pathways are similar to the previous cases. Figure 4 shows the RS flowchart. This algorithm shares many similarities with *FRS,* except that the “a priori” sampling distribution is uniform in this case. This algorithm works with smaller amount of prior information that makes it more explorative than FRS.

#### Bayesian networks (BNs)

A *BN* is a data structure that encodes the conditional probability distribution between variables by using a directed acyclic graph. This procedure is utilized to sample the posterior distribution of the genetic signatures, P(**g**/**c**^obs^), according to Bayes rule (2) .

**Fig. 5:**
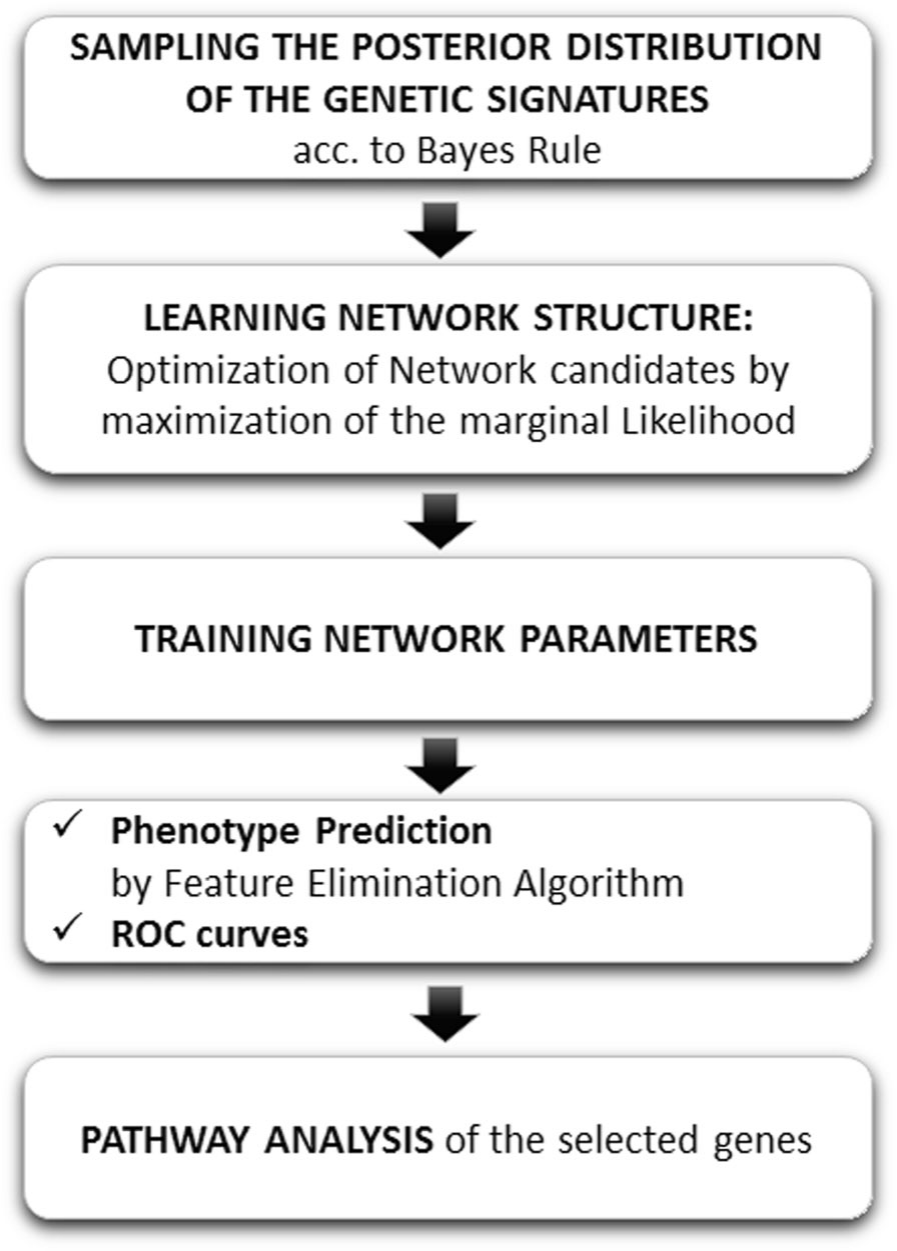
Bayesian Network workflow

In reality, this algorithm carries out the gene selection in two steps (Fig. 5). First, the training dataset is used to “learn” the network structure. The best network model is determined by selecting the candidate network model with the highest computed marginal likelihood. The “learned” network exemplifies how genes affects each other and serves as a phenotype predictor. At the second step, the network parameters are trained by optimizing the conditional probabilities of the network. Finally, the phenotype prediction is carried out using a variable elimination algorithm [22]. The genes associated to the final *BN* are used to identify the detective pathways [10–13].

CGBayesNets was used to accomplish the *BNs* modelling [12] and maximizing the data likelihood. Since the number of possible networks grows exponentially with the number of gene candidates, not all networks can be explored, and different heuristics are employed to optimize this search. Therefore, this algorithm cannot be considered as a pure sampling algorithm. In fact, exploring the uncertainty space of the high discriminatory *BNs* is more advantageous, in terms of pathway analysis, since the BN found is not an unique illustration of the phenotype problem uncertainty, that is, other plausible networks might exist that explain the phenotype with a similar likelihood. Besides, the noise in data and class assignment [2] falsifies the pathways analysis and greatly affects the BNs search and optimization. Therefore, inferring the genetic pathways via the genes involved in the best network might not be robust enough.

### Identification of the altered genetic pathways

GeneAnalytics [16] was used to infer the defective pathways and biological processes by querying the group of genes with the higher sampling frequency for these sampling algorithms. This software uses the main ontological databases (Biosystems, Reactome, Qiagen, Kegg, Cell Signaling Technology and R&D Systems), and offers important information about the chemo-biology behind the phenotypic expressions of actionable genes. Furthermore, it also provides information about the chemical compounds to target the actionable genes that characterize the phenotype. In all the cases, this analysis was performed over the high discriminatory networks of genes, provided by each sampler.

## Results

Additional file 1: Tables S1 and S2 show these lists of the first most discriminative genes, ranked by their Fisher’s ratio and the corresponding accuracies of each list, for both prediction problems, providing also the means, standard deviations and Fold change of the expressions of genes in each class, together with accuracies corresponding to each list. In the case of metastasis prediction problem the small-scale genetic signature found by the recursive backward feature elimination [15] was composed of the 92 most discriminatory genes (Additional file 1: Table S1) with an LOOCV predictive accuracy of 96.3%. In the case of survival prediction (Additional file 1: Table S2) this small-scale signature contained only 16 genes with 94.4%. These predictive accuracies were respectively improved to 98 and 96.3% by the Fisher’s ratio sampler.

Tables 1 and 2 show the most frequent genes involved in the high discriminative signatures provided by each sampling algorithm for Metastasis and Survival prediction. The frequencies are established over the total amount of sampled genes within the high predictive networks that have an accuracy greater than 85% found after 10^5^ random simulations. All the sampling algorithms, but BNs, are very fast. These simulations were performed in less than 5 min in a regular computer. Tables 3 and 4 present the list of the most frequently pathways sampled by each algorithm together with their relative score.

**Table 1:** Metastasis prediction: list of most-frequently sampled genes by the different algorithms

FRS	HS	RS	BN
**LINC00630**	OTUB2	**LINC00630**	ZNF597
**LOC100506272**	**STC1**	HIPK3	ZDHHC2
**STC1**	**BAIAP2-AS1**	CCDC116	YY1
**BAIAP2-AS1**	KCNS2	**EXOC5**	SPP1
**ARFGAP2**	**LOC100506272**	GHSR	SMAD9
LHX9	LOC644135	ZNF540	SHANK1
**LOC646482**	**LINC00630**	ATF3	RBMS3
CACNA1S	UGT1A1	1557882_at	PRICKLE1
AC108056.1	**ARFGAP2**	220899_at	PRDM11
NXF3	CACNA1I	**ARFGAP2**	PML
GIPC3	DCAF8	CXADR	NAV1
KCNS2	RP11-799D4.4	AHI1	MASP1
DAZ1	MDM2	KIRREL3-AS3	**LOC646482**
UGT1A1	RP11-38C18.3	DRP2	LOC101927735
RP5-855D21.1	BFSP2-AS1	207743_at	P4HA2
**EXOC5**		JMJD6	LINC00642

Bold faces highlights the common genes

**Table 2:** Survival prediction: list of the most-frequently sampled genes by the different algorithms

FRS	HS	RS	BN
**LOC100506272**	**LOC100506272**	ING2	ZNF658
EML3	CHAF1A	220899_at	LIMK2
TYR	LOC400748	LINC00423	HAPLN2
ABCB8	KCNS2	VSX1	237969_at
GYPA	ZNF428	1561100_at	LOC644135
C14orf80	DAZ1	1558494_at	240923_at
LILRA2	**LOC646482**	LOC100507530	ANKRD54
1564841_at	**LINC00630**	LINC01020	UBN2
RP11-440I14.2	233714_at	206909_at	234834_at
LATS2	TNRC18	C2CD3	215828_at
241286_at	1558494_at	1566162_x_at	CACNG8
LOC100506411	DNASE1L3	CXADR	CTSC
PTPN21	RP11-38C18.3	213777_s_at	EPS15P1
UPF3A	PCDHB2	PRKCB	HCN2
RGSL1	DCAF8	240973_s_at	P2RX5-TAX1BP3
232723_at	ME1	BTG4	MMP14

Bold faces highlights the common genes

**Table 3:** Metastasis prediction: top score pathways sampled by the different algorithms

FRS		HS	
Score	Top Pathways	Score	Top Pathways
10.3	**Direct P53 Effectors**	11.2	**JNK Signaling in CD4+ TCR Pathway**
10.1	**DREAM Repression & Dynorphin Exp.**	9.7	**RhoA Signaling Pathway**
9.6	**P53 Signaling**	8.3	ATM Pathway
8.8	**RhoA Signaling Pathway**	8.1	FoxO Signaling Pathway
8.4	**P53 Pathway**	8.0	**TGF-beta Signaling Pathway**
**RS**		**BN**	
**Score**	**Top Pathways**	**Score**	**Top Pathways**
15.0	**DREAM Repression & Dynorphin Exp.**	8.1	**Direct P53 Effectors**
10.1	**Direct P53 Effectors**	8.0	Proteolysis Putative SUMO-1 Pathway
10.1	Immune Response Role of DAP12 Receptors in NK Cells	7.1	Creation of C4 and C2 Activators
9.9	**JNK Signaling in CD4+ TCR Pathway**	6.9	**TGF-beta Receptor Signaling**
9.8	MAPK Signaling Pathway	6.8	MTOR Signaling Pathway

Bold faces highlights the common pathways

**Table 4:** Survival prediction: top score pathways sampled by the different algorithms

FRS		HS	
Score	Top Pathways	Score	Top Pathways
9.87	**Integrin Pathway**	13.54	**Integrin Pathway**
8.96	Fatty Acid Beta-oxidation (peroxisome)	11.30	**Sweet Taste Signaling**
7.94	**DREAM Repression &Dynorphin Expression**	11.28	**DREAM Repression &Dynorphin Expression**
7.88	**Signaling Events Mediated By HDAC Class II**	11.13	RhoA Signaling Pathway
7.54	Type II Interferon Signaling (IFNG)	9.58	**Signaling Events Mediated By HDAC Class II**
7.19	Fatty Acid Biosynthesis (KEGG)	9.40	Androgen Receptor Signaling Pathway
6.93	Fatty Acyl-CoA Biosynthesis	9.39	CCR5 Pathway in Macrophages
**RS**		**BN**	
**Score**	**Top Pathways**	**Score**	**Top Pathways**
9.90	TCR Signaling	7.87	Nucleotide-binding Domain, NLR Signaling
9.79	Androgen Receptor Signaling Pathway	7.39	Apoptosis and Autophagy
9.48	Presenilin-Mediated Signaling	7.20	C-MYC Transcriptional Repression
8.57	Ovarian Infertility Genes	6.82	NF-kB (NFkB) Pathway
8.34	DNA Damage Response (ATM Dependent)	6.19	Apoptosis Modulation and Signaling
8.14	Apoptotic Pathways in Synovial Fibroblasts	6.13	Apoptosis and Survival Caspase Cascade
8.02	**Sweet Taste Signaling**	5.69	Senescence and Autophagy in Cancer

Bold faces highlights the common pathways

**Fig. 6:**
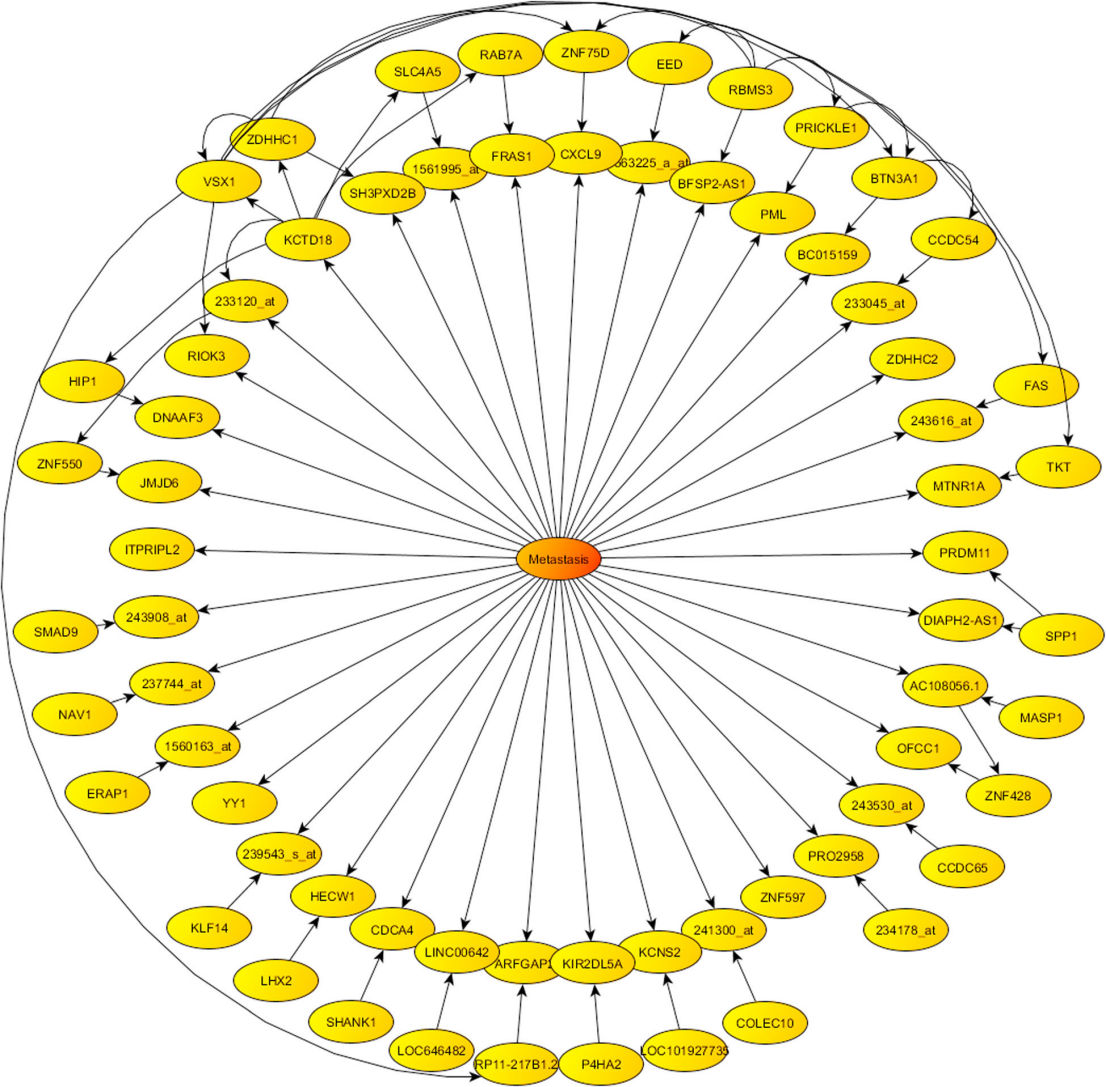
Metastasis prediction: optimum centered Bayesian network found

**Fig. 7:**
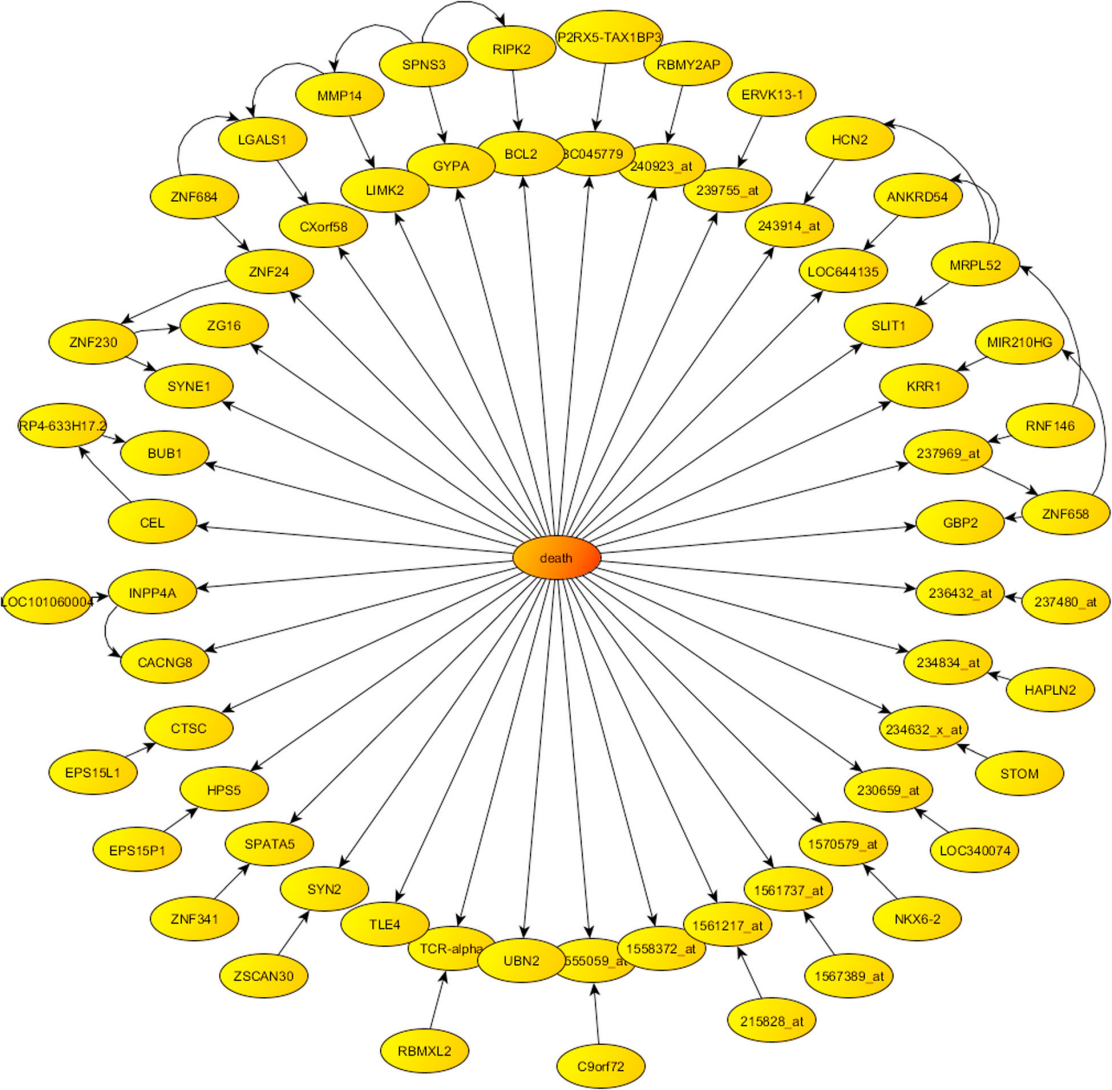
Survival prediction: optimum centered Bayesian network found

Figures 6 and 7 show the optimum Bayesian Networks found for the TNBC metastasis and survival phenotype prediction problem, containing respectively 68 and 66 genes. As it has been already mentioned, it is important to highlight that this probabilistic factorization of the uncertainty space in both phenotype prediction problems (metastasis and survival) is not unique.

## Discussion

The aim of this research was to appraise the abilities of three novel sampling algorithms to predict phenotypic changes using data from patients with TNBC and to determine if any or all of these algorithms was equivalent or superior to BN-based methods. We were able to identify different discriminatory networks of the phenotype to infer the altered biological pathways. Underlying this analysis, we would like to demonstrate the hypothesis of biological invariance, that is, the defective pathways that affect the disease development should be somehow independent.

### Metastasis prediction

Some of the most frequently sampled genes shown in Table 1 for the metastasis prediction are shared by the Fisher’s ratio, Holdout and Random Samplers. In contrast, this finding was not the case for the Bayesian Network that only choses one of possible probabilistic factorization of the metastasis phenotype prediction. The main objective is to understand how the most important genes work in synergy; however, the individual ontological attributions of the most-discriminatory genes in the prediction of the TNBC phenotype are very important to understand the pedigree of these genes.

All the genes provided by FRS are overexpressed in the metastasis group, and the most frequent sampled gene was LINC00630. Mao et al. [23] studied the role of non-coding RNAs showing that LINC00630 play a crucial role in the development of Non-Small-Cell Lung Cancers. It was also proved that its overexpression increased cell proliferation and metastasis in vitro and in vivo whereas LINC00630 silencing had opposite effects. Therefore, LINC00630 constitutes a very interesting target. STC1 (Stanniocalcin-1) encodes a glycoprotein that is expressed in a wide diversity of tissues. Overexpression of STC1 in mice produces high serum phosphate levels, dwarfism and increased metabolic rate. This gene has also an altered expression in hepatocellular, ovarian and breast cancers and it has been previously associated to metastasis in TNBC [24, 25]. BAIAP2-AS1 is a non-coding RNA gene. This gene has been associated to hepatitis B virus-related hepatocellular carcinoma [26]. Metabolism related and cancer associated KEGG pathways are in relation with BAIAP2-AS1. As well, it has been found that BAIAP2-AS1 may function as a competing endogenous RNA (ceRNA), regulating other RNA transcripts. ARFGAP2 (ADP Ribosylation Factor GTPase Activating Protein 2) is a Protein coding gene that it is involved in protein recycling (Transport of the damaged proteins to the Golgi and subsequent modification). LHX9 is a gene involved in transcription.

Similarly, in the case of HS, all high frequently sample genes, except LOC644135, are overexpressed in the metastasis group. The most important gene seems to be STC1 (Stanniocalcin-1) with 2 different probes in the set of most important sampled genes. Other important gene is OTUB2 that codes one enzyme, which is required to reverse the ubiquitin modification of deleterious proteins. Among its related pathways are ovarian tumor domain proteases and protein ubiquitination. HS also sampled BAIAP2-AS1 as a high frequent gene. Another appraised gene is KCNS2, which encodes a protein that is a voltage-gated potassium channel subunit. Huang and Yeh Jan have reviewed the importance of potassium channels in regulating cancer cell migration and proliferation [27]. The potassium channel activation inhibits proliferation of breast cancer cells [28]. LOC100506272 and LOC644135 are two uncharacterized genes.

The main genes found by RS have the following attributions: HIPK3 encodes a serine/threonine-protein kinase, which takes part in the transcription regulation and apoptosis. CCDC116 (coiled-coil domain containing 116) is primarily found in the testis. This gene has been recently connected to risk in multiple kind of cancers [29] and it is considered in experimentation as a possible prostate cancer biomarker. EXOC5 is related to peptide hormone metabolism. GHSR is the Growth Hormone Secretagogue Receptor and is related to the CAMP signaling pathway.

In the case of BNs, the main genes are related to the TGF-beta Receptor Signaling and MTOR Signaling Pathway. ZNF597 codes a zinc finger protein, which is involved in gene expression and transcription. ZDHHC2 (Palmitoyltransferase or Reduced Expression Associated with Metastasis Protein) has been linked to human colorectal cancers with liver metastasis [30]. Yin Yang 1 (YY1) is vastly expressed in several sorts of cancers and regulates tumorigenesis through numerous pathways. YY1 is overexpressed in breast cancer cells [31]. Genes found by BN are different from those found by other sampling methods. This observation seems most to be attributable to the high-underdetermined character of the phenotype prediction problem. Therefore, using the optimum probabilistic network found is not the proper way of spanning the phenotype prediction uncertainty.

Comparing the different lists, the most frequently sampled gene, LINC00630, is the same in FRS and RS. HS sampled this gene with a lower frequency. Other common genes (sampled by all algorithms) were STC1, LOC100506272, BAIAP2-AS1 and LOC646482.

The analysis of the top score pathways shown in Table 2 provides the following insights:

- Fisher’s ratio and Random samplers found as the main common mechanisms P53 pathways and DREAM Repression and Dynorphin Expression.
- Fisher’s ratio and Holdout samplers shared the RhoA Signaling Pathway.
- Holdout and Random samplers have JNK Signaling in CD4+ TCR Pathway in common.
- Bayesian networks share P53 pathways (even thou with a lower score) with Fisher’s ratio and Random Samplers, and TGF-beta Receptor Signaling with Holdout sampler. The Bayesian network used the network with fewer discriminatory genes (only 68). This fact influences the pathway identification. However, Direct P53 effectors and TGF-beta receptor signaling pathways appeared also related to other samplers. The main biological process involved is the Complement Activation via the Lectin pathway. The pathways and biological processes identified by Bayesian networks have a lower score than those found by the rest of the samplers.
- In all the cases, the pathways are involved in both cancer and immune response. Despite not being the purpose of this paper, it is very remarkable to mention that the primary biological process involved is phagocytosis, which is one of the most important mechanisms in the immune system defense related to NF-κB activation.

### Survival prediction

The most frequently sampled genes shown in Table 2 for the survival prediction are very different for all the samplers. Only LOC100506272 (uncharacterized gene) is in common to *FRS* and *HS* within this very restrictive list. Although not shown in Table 2, *RS* also sampled this gene with a lower frequency (0.15%). Besides, LINC00630 is a common for all the algorithms in lower positions. Interesting, this gene resulted as the most frequent gene in the metastasis prediction. Other high frequent genes in the metastasis prediction problem are also present in the survival prediction (even thou with lower frequencies that are not shown in Table 2), such as BAIAP2-AS1, described before, sampled by *HS* algorithm, LOC646482, STC1 and ZNF597 sampled by RS and HS. Other common genes sampled by all algorithms for survival prediction problem were EML3, TYR, ABCB8 and GYPA. To our knowledge, none of these genes was previously associated to breast cancer. *HS* and *RS* sampled other common genes such as CHAFIA, LOC400748, KCNS2, ZNF428, ING2, LINC00423 and VSX1. Among these genes, ING2 (Inhibitor Of Growth Family Member 1) encodes a tumor suppressor protein that can induce cell growth arrest and apoptosis, responsible of biological process such as Regulation of Cell Death and Protein Import into Nucleus, and, related to Squamous Cell Carcinoma, Head And Neck, Fibrosarcoma of Bone, Squamous Cell Carcinoma, and Melanoma diseases.

The high heterogeneity observed in these lists implies that there are many genetic networks that equally predict survival in TNBC, that is, the uncertainty space of this problem is broader than the one corresponding to the metastasis prediction. This fact can be observed also in the analysis of the top score pathways where only *FRS* and *HS* commonly sampled with high score the Integrin Pathway, the DREAM Repression and Dynorphin Expression, and the Signaling Events Mediated by HDAC Class II. The role of integrin signaling in breast cancer has been highlighted by Lambert et al. [32]. The extracellular matrix which is composed of numerous insoluble proteins secreted locally by epithelial and stromal cells changes dramatically during the process of breast tumorigenesis and can strongly affect disease progression [33]. Interesting, the DREAM Repression and Dynorphin Expression pathway was found to be the top score mechanism involved in TNBC metastasis.

## Conclusions

In this paper, we compared three novel samplers in phenotype prediction problems (Fisher’s ratio, Holdout and Random Samplers) with Bayesian Networks to unravel the altered genetic pathways involved in the metastasis and survival in Triple Breast Negative Cancer. Among them, the Bayesian networks is the less efficient methodology, since it requires a greater computational effort to find out the discriminatory networks. This research shows that the Fisher’s ratio, Holdout and the Random samplers are good alternatives to Bayesian networks, since they are much more efficient when sampling the uncertainty space of the phenotype prediction problem. These algorithms are based in a different paradigm and they do not require inferring the posterior probability distribution. Besides, the pathways found by these novel samplers explained better mechanistically this disease. This analysis shows that the common pathways in TNBC metastasis and survival are related to cancer progression and immune response, although the matching with the results obtained via Bayesian networks was not perfect. This result could be improved by considering other Bayesian Networks with lower predictive accuracy, since different equivalent probabilistic factorizations exist. Besides, 70 genes are not enough to establish mechanistic conclusions about the disease development and prognosis, as shown by the complexity of TNBC (high number of discriminatory genes). Conversely, the right way of performing this analysis consists in sampling different discriminatory networks of the phenotype and using the most-frequently sampled genes to establish the defective pathways. Damping the effect of the helper genes that have a lower discriminatory power of the phenotype and whose importance mechanistically is much lower is crucial to perform a robust sampling of the defective pathways. The appearance of these genes in the pathway analysis is mainly related to the high-underdetermined character of the phenotype prediction. This approach is similar to the Lasso regularization in inversion [34]. These sampling approaches are more robust than the Bayesian Networks, since the probabilistic representation (acyclic graph) corresponding to the uncertainty space of the phenotype prediction (for a given classifier) is not unique. Therefore, the best BN, which is found through optimization, cannot considered a robust way to sample the defective pathways involved in a phenotype prediction problem. This would equivalent to trying to understand the genetic mechanisms involved in this disease by using the discriminatory network with the higher predictive accuracy, which would be very sensible to the presence of noise in the expression data, and mainly in the class assignment. This research also confirms our prior insight that the altered pathways should be somehow independent of the sampling methodology that is used to infer them, and it is crucial important to understand the discriminatory power of a gene and how genetic networks work in synergy. Further research will be devoted in the future to analyze this important subject in order to finally prove the hypothesis of biological invariance. Although the methodology shown in this paper has a general purpose, we hope that the results shown for TNBC in the discussion concerning novel pathways and genetic targets, will contribute to provide deeper insights into their cure.

## Supplementary information

**Additional file 1.** Metastasis and Survival prediction.

## Data Availability

The dataset used in this paper can be accessed in the GEO database under the acronym GSE58812.

## Abbreviations

BN: Bayesian Network
CCPPRB: French Committee for the Protection of Human Subjects
FRS: Fisher’s Ratio Sampler
GEO: Gene Expression Omnibus
HS: Holdout Sampler
LOOCV: Leave-One-Out-Cross-Validation
NGS: Next Gen Sequencing
RS: Random Sampler
TNBC: Triple Negative Breast Cancer
