# Supplementary material for: Robust pathway sampling in phenotype prediction. Application to triple negative breast cancer
Source: BMC Bioinformatics. 2020 Mar 11;21(Suppl 2):89. doi: 10.1186/s12859-020-3356-6 (PMC7068866; doi:10.1186/s12859-020-3356-6)
Supplement: Supplementary file 1 — Additional file 1. Metastasis and Survival prediction. [file 12859_2020_3356_MOESM1_ESM.docx]

**Supplementary Material. Metastasis and Survival prediction**

**Table 1S.** Metastasis prediction: most discriminatory genes ordered by their Fisher’s ratio.

| **GeneName** | **MeanC1** | **StdC1** | **MeanC2** | **StdC2** | **FC** | **FR** | **Accuracy** |
| --- | --- | --- | --- | --- | --- | --- | --- |
| LINC00630 | 8 | 8 | 15 | 10 | -0.84 | 1.12 | 70.09 |
| LOC100506272 | 16 | 17 | 23 | 13 | -0.5 | 0.9 | 72.9 |
| STC1 | 263 | 165 | 382 | 171 | -0.54 | 0.72 | 79.44 |
| BAIAP2-AS1 | 16 | 12 | 24 | 13 | -0.53 | 0.67 | 72.9 |
| GIPC3 | 38 | 35 | 60 | 43 | -0.66 | 0.66 | 80.37 |
| 1561834_a_at | 33 | 24 | 49 | 27 | -0.6 | 0.66 | 80.37 |
| CACNA1I | 38 | 26 | 57 | 24 | -0.59 | 0.65 | 82.24 |
| KCNS2 | 16 | 17 | 27 | 16 | -0.7 | 0.62 | 85.98 |
| 1558905_at | 12 | 9 | 9 | 9 | 0.55 | 0.6 | 86.92 |
| NXF3 | 15 | 16 | 23 | 16 | -0.65 | 0.59 | 84.11 |
| RP11-38C18.3 | 21 | 17 | 30 | 19 | -0.52 | 0.59 | 84.11 |
| ARFGAP2 | 22 | 17 | 31 | 18 | -0.52 | 0.58 | 85.05 |
| LOC646482 | 24 | 17 | 32 | 18 | -0.42 | 0.58 | 85.05 |
| MDM2 | 20 | 14 | 28 | 13 | -0.51 | 0.58 | 82.24 |
| UGT1A1 | 26 | 19 | 39 | 17 | -0.59 | 0.56 | 86.92 |
| LHX9 | 15 | 13 | 19 | 11 | -0.38 | 0.56 | 87.85 |
| ANKRD54 | 558 | 136 | 682 | 206 | -0.29 | 0.53 | 87.85 |
| DAZ1 /// DAZ2 /// DAZ3 /// DAZ4 | 10 | 9 | 16 | 12 | -0.67 | 0.53 | 86.92 |
| 216864_at | 12 | 13 | 18 | 14 | -0.55 | 0.53 | 85.98 |
| AC108056.1 | 13 | 12 | 17 | 11 | -0.46 | 0.53 | 88.79 |
| RP5-855D21.1 | 19 | 13 | 30 | 19 | -0.67 | 0.51 | 87.85 |
| 233502_at | 17 | 10 | 12 | 11 | 0.49 | 0.51 | 88.79 |
| AJ606316 /// LOC644135 | 26 | 21 | 14 | 16 | 0.88 | 0.51 | 89.72 |
| NIN | 1539 | 564 | 1205 | 427 | 0.35 | 0.5 | 90.65 |
| C2CD3 | 33 | 22 | 46 | 20 | -0.49 | 0.5 | 89.72 |
| 233714_at | 10 | 10 | 17 | 12 | -0.74 | 0.49 | 90.65 |
| RP11-799D4.4 | 25 | 16 | 15 | 12 | 0.78 | 0.49 | 93.46 |
| SOX4 | 10514 | 4895 | 13768 | 5688 | -0.39 | 0.48 | 92.52 |
| OTUB2 | 29 | 23 | 45 | 30 | -0.66 | 0.48 | 94.39 |
| KDM5B | 14 | 10 | 21 | 13 | -0.57 | 0.48 | 93.46 |
| BC037214 /// RP11-58G13.1 | 10 | 11 | 16 | 13 | -0.69 | 0.48 | 93.46 |
| N4BP2L1 | 1548 | 705 | 1115 | 517 | 0.47 | 0.48 | 93.46 |
| 1561670_at | 14 | 12 | 23 | 17 | -0.73 | 0.48 | 93.46 |
| RP4-621B10.8 | 11 | 9 | 15 | 10 | -0.42 | 0.47 | 93.46 |
| COLEC10 | 20 | 19 | 28 | 18 | -0.43 | 0.47 | 93.46 |
| DCAF8 | 26 | 20 | 40 | 26 | -0.63 | 0.46 | 93.46 |
| EXOC5 | 10 | 9 | 15 | 8 | -0.53 | 0.46 | 94.39 |
| 1565732_at | 26 | 19 | 18 | 17 | 0.52 | 0.46 | 92.52 |
| RBM47 | 18 | 16 | 24 | 16 | -0.44 | 0.46 | 92.52 |
| ZC2HC1B | 24 | 17 | 17 | 13 | 0.52 | 0.45 | 92.52 |
| RP3-333B15.4 | 48 | 22 | 66 | 27 | -0.47 | 0.45 | 92.52 |
| TMEM165 | 22 | 14 | 15 | 11 | 0.56 | 0.45 | 93.46 |
| ST8SIA5 | 26 | 18 | 38 | 18 | -0.54 | 0.45 | 91.59 |
| 241052_at | 12 | 12 | 19 | 14 | -0.65 | 0.45 | 93.46 |
| NCS1 | 116 | 48 | 161 | 58 | -0.47 | 0.44 | 93.46 |
| LIMK2 | 813 | 373 | 1046 | 386 | -0.36 | 0.44 | 92.52 |
| LOC284669 | 18 | 16 | 24 | 17 | -0.44 | 0.44 | 92.52 |
| DNAH1 | 19 | 15 | 32 | 19 | -0.73 | 0.44 | 94.39 |
| C4orf33 | 324 | 165 | 240 | 185 | 0.44 | 0.44 | 92.52 |
| PRDM11 | 22 | 17 | 32 | 24 | -0.51 | 0.43 | 92.52 |
| KIAA0485 | 51 | 22 | 65 | 27 | -0.34 | 0.43 | 92.52 |
| HNRNPM | 21 | 16 | 28 | 17 | -0.42 | 0.43 | 92.52 |
| CXADR | 4070 | 2967 | 5891 | 3043 | -0.53 | 0.43 | 92.52 |
| RNF215 | 31 | 26 | 39 | 23 | -0.35 | 0.43 | 94.39 |
| STC1 | 723 | 533 | 1182 | 677 | -0.71 | 0.42 | 92.52 |
| TKT | 4391 | 1910 | 5573 | 1830 | -0.34 | 0.42 | 92.52 |
| MCTS1 | 28 | 15 | 22 | 15 | 0.37 | 0.42 | 93.46 |
| LOC51145 | 20 | 14 | 16 | 16 | 0.36 | 0.42 | 93.46 |
| BC043227 | 8 | 8 | 13 | 10 | -0.69 | 0.42 | 91.59 |
| PHLDB2 | 12 | 13 | 24 | 19 | -1.02 | 0.42 | 90.65 |
| 213777_s_at | 32 | 16 | 19 | 13 | 0.75 | 0.41 | 93.46 |
| 233239_at | 122 | 39 | 93 | 39 | 0.39 | 0.41 | 92.52 |
| FRAS1 | 33 | 26 | 42 | 28 | -0.33 | 0.41 | 91.59 |
| 237348_at | 40 | 30 | 50 | 30 | -0.32 | 0.41 | 91.59 |
| IPO5P1 | 12 | 11 | 16 | 11 | -0.38 | 0.41 | 91.59 |
| 1560800_at | 8 | 8 | 11 | 8 | -0.42 | 0.41 | 92.52 |
| 234160_at | 16 | 16 | 22 | 17 | -0.46 | 0.4 | 91.59 |
| 240324_at | 103 | 45 | 80 | 35 | 0.36 | 0.4 | 90.65 |
| SLC7A8 | 26 | 17 | 43 | 25 | -0.74 | 0.4 | 92.52 |
| 233045_at | 18 | 13 | 27 | 14 | -0.62 | 0.4 | 91.59 |
| RNF125 | 895 | 481 | 578 | 291 | 0.63 | 0.4 | 91.59 |
| 238265_x_at | 73 | 33 | 52 | 26 | 0.48 | 0.4 | 92.52 |
| HAPLN2 | 30 | 19 | 41 | 23 | -0.41 | 0.39 | 92.52 |
| FAM19A1 | 25 | 17 | 15 | 12 | 0.76 | 0.39 | 93.46 |
| HELQ | 40 | 26 | 25 | 21 | 0.69 | 0.39 | 92.52 |
| 240973_s_at | 28 | 16 | 19 | 15 | 0.53 | 0.39 | 93.46 |
| 216163_at | 25 | 15 | 19 | 14 | 0.45 | 0.39 | 92.52 |
| BDH2 | 2340 | 967 | 1877 | 744 | 0.32 | 0.39 | 93.46 |
| KRT8 | 2897 | 2263 | 4465 | 2274 | -0.62 | 0.38 | 93.46 |
| 1561995_at | 27 | 25 | 37 | 24 | -0.42 | 0.38 | 93.46 |
| TMEM53 | 360 | 144 | 456 | 159 | -0.34 | 0.38 | 93.46 |
| KIAA2022 | 45 | 21 | 32 | 20 | 0.51 | 0.38 | 93.46 |
| ZNF550 | 21 | 12 | 16 | 15 | 0.43 | 0.38 | 92.52 |
| LINC00944 | 21 | 12 | 28 | 14 | -0.39 | 0.38 | 93.46 |
| SGPP1 | 293 | 141 | 225 | 127 | 0.38 | 0.37 | 92.52 |
| TOR1AIP2 | 110 | 38 | 136 | 46 | -0.3 | 0.37 | 92.52 |
| 237508_at | 7 | 6 | 10 | 8 | -0.63 | 0.37 | 93.46 |
| BFSP2-AS1 | 16 | 16 | 24 | 18 | -0.53 | 0.37 | 92.52 |
| LOC102723645 | 12 | 11 | 16 | 12 | -0.42 | 0.37 | 94.39 |
| 241492_at | 31 | 14 | 21 | 12 | 0.57 | 0.37 | 93.46 |
| ZNF540 | 46 | 27 | 29 | 22 | 0.66 | 0.37 | 94.39 |
| CXADR | 2180 | 1637 | 3206 | 1681 | -0.56 | 0.37 | 96.26 |

**Table 2S.** Survival prediction: most discriminatory genes ordered by their Fisher’s ratio. C1 stands for patients that survived during the studied period and C2 for samples associated with exitus.

| **GeneName** | **MeanC1** | **StdC1** | **MeanC2** | **StdC2** | **FC** | **FR** | **Accuracy** |
| --- | --- | --- | --- | --- | --- | --- | --- |
| LOC100506272 | 16 | 16 | 24 | 13 | -0.58 | 0.93 | 68.22 |
| LINC00630 | 9 | 8 | 14 | 10 | -0.75 | 0.88 | 71.96 |
| 1558494_at | 14 | 13 | 23 | 15 | -0.66 | 0.83 | 76.64 |
| KCNS2 | 16 | 15 | 30 | 20 | -0.91 | 0.77 | 77.57 |
| LOC400748 | 13 | 12 | 20 | 12 | -0.59 | 0.73 | 78.5 |
| PCDHB2 | 905 | 905 | 1360 | 939 | -0.59 | 0.7 | 77.57 |
| DAZ1 | 10 | 9 | 16 | 12 | -0.74 | 0.68 | 83.18 |
| ZNF428 | 35 | 22 | 53 | 21 | -0.59 | 0.68 | 81.31 |
| TNRC18 | 13 | 12 | 22 | 15 | -0.72 | 0.67 | 86.92 |
| CHAF1A | 20 | 18 | 29 | 15 | -0.48 | 0.66 | 87.85 |
| GIPC3 | 38 | 35 | 61 | 44 | -0.68 | 0.65 | 88.79 |
| LOC646482 | 23 | 17 | 34 | 19 | -0.56 | 0.62 | 90.65 |
| DNASE1L3 | 33 | 25 | 19 | 20 | 0.78 | 0.58 | 90.65 |
| 233714_at | 10 | 9 | 18 | 12 | -0.79 | 0.58 | 91.59 |
| ME1 | 20 | 23 | 36 | 25 | -0.81 | 0.57 | 92.52 |
| ITPRIPL2 | 19 | 16 | 27 | 15 | -0.47 | 0.57 | 94.39 |
